# Expert consensus on relevant topics for undergraduate paediatric dental curriculum using the fuzzy Delphi method: a new direction for Malaysian dental education

**DOI:** 10.1186/s12903-023-03130-8

**Published:** 2023-07-05

**Authors:** Galvin Sim Siang Lin, Yu Jie Chin, Rob Son Chong, Fadzlinda Baharin, Sharifah Wade’ah Wafa Syed Saadun Tarek Wafa, Nabihah Dziaruddin

**Affiliations:** 1 Department of Dental Materials, Faculty of Dentistry, Asian Institute of Medicine, Science and Technology (AIMST) University, 08100 Kedah, Malaysia; 2 Department of Paediatric Dentistry and Orthodontics, Faculty of Dentistry, Universiti Malaya, Kuala Lumpur, 50603 Malaysia; 3 Paediatric Dentistry Unit, School of Dental Sciences, Universiti Sains Malaysia, Health Campus, 16150 Kelantan, Malaysia; 4 AVISENA Women’s & Children’s Specialist Hospital, 40000 Selangor, Malaysia

**Keywords:** Paediatric dentistry, Delphi technique, Dental caries, Dental education, Undergraduate

## Abstract

**Background:**

Paediatric dentistry is a branch of dental specialty that focuses on dental care for children from infancy through adolescence. However, there is no standardised national undergraduate paediatric dental curriculum in Malaysia. The present study aimed to identify relevant topics for undergraduate paediatric dental curricula and to determine the appropriate cognitive and psychomotor levels for each topic based on the consensus among paediatric dental experts.

**Methods:**

Potential relevant undergraduate paediatric dentistry topics were initially drafted and revised according to the revised national competency statement. The final draft included 65 topics clustered under 18 domains. A fuzzy Delphi method was used and experts who fulfilled the inclusion criteria were invited to anonymously ranked the importance of relevant topics using a five-point Likert scale and proposed suitable cognitive and psychomotor levels for each topic. Fuzzy evaluation was then performed, and experts were considered to have reached a consensus if the following three conditions were achieved: (a). the difference between the average and expert rating data was ≤ 0.2; (b). the average expert consensus was ˃70%; and (c). the average fuzzy number was ≥ 0.5. Subsequently, the mean ratings were used to determine the cognitive and psychomotor levels.

**Results:**

20 experts participated in the survey. 64 out of 65 paediatric dentistry topics were deemed acceptable. The average fuzzy number ranged from 0.36 to 0.85, while the average Likert score ranged from 3.05 to 5.00. The topic “Dental amalgam” was rejected based on expert consensus since the average fuzzy number was 0.36. The most significant topic was “Pit and fissure sealant”, followed by “Preventive advice”, “Early childhood caries”, “Dental caries in children & adolescent”, “Management of dental caries in paediatric patients”, and “Consent” which were equally ranked as the second most important topics. According to Bloom’s and Simpson’s taxonomies, most of the paediatric dentistry topics were rated adequate for undergraduate students at the cognitive level of “Apply” (C3) and a psychomotor level of “Guided response” (P3).

**Conclusion:**

The current study successfully identified relevant undergraduate paediatric dentistry topics using the fuzzy Delphi method, which can facilitate future educators to improve existing Malaysian undergraduate paediatric dental curricula.

## Background

Malaysia has 13 dental schools spread across the country, each of which offers a five-year undergraduate dental programme [1]. With Bloom’s cognitive and Simpson’s psychomotor taxonomies serving as the basis for establishing dental curricula, undergraduate dental programmes in Malaysia are organised into preclinical and clinical phases [2]. During the preclinical phase, students are introduced to basic medical and dental principles, as well as operative practical and laboratory skills, while the clinical phase of the programme allows students to provide patient care in the dental clinic under close supervision [3]. The Malaysian Dental Council (MDC) is entrusted with regulating and governing the quality assurance of undergraduate dental programmes [4], as well as recognising dental qualifications for registration of practitioners under the Malaysian Dental Act 2018 [5]. This is done to keep Malaysia’s dental education and training at a high level. Additionally, the Malaysian Qualifications Agency (MQA) contributes to the accreditation of each dental school and the provision of quality assurance concerning the school’s compliance with the minimal standards of fundamental dental education and training [5].

Paediatric dentistry is a branch of dental speciality that deals with dental care for children from infancy through adolescence [6]. Undergraduate paediatric dental education in Malaysia provides all facets of dental treatment for children which includes preventive and restorative procedures. Unfortunately, there is no uniformity and standardisation for a national paediatric dental curriculum in Malaysia, and all dental schools operate independently as compartmentalised institutions. Malaysian undergraduate dental programmes are separated into preclinical and clinical phases [3]. Dental students spend two years in the preclinical phase learning the fundamentals of medicine and dental sciences before moving on to the clinical phase, in which they need to spend the next three years providing patient care in clinics under supervision [7]. Paediatric dentistry is introduced at some dental schools during the preclinical phase of undergraduate curricula, whereas in others it is introduced later during the clinical phase. Furthermore, there is no consensus among dental schools as to the topics that should be covered in the undergraduate curriculum for paediatric dentistry. Due to the lack of such standards, undergraduate paediatric dental education in Malaysia is likely to vary considerably, creating a dental workforce with a diverse range of knowledge and skills [8]. It is pivotal that dental schools should prepare their future graduates to be competent in managing common oral health diseases for infants, children, adolescents, and paediatric patients with special care needs [8, 9].

The Malaysian Dental Dean Council has organised a workshop in June 2021 and proposed a revision to the national dental graduate’s competency statements, highlighting a transition towards competency-based dental education [10, 11]. Among the desired cognitive and psychomotor-related clusters for paediatric dentistry, it has been stated that future dental graduates should be able to differentiate the principles of restoration and replacement of primary and permanent dentition (Bloom’s cognitive level of C4: Analyse – able to differentiate between components and relate them to each other and overall structure), perform simple restorative procedures in primary and permanent dentition (Simpson’s psychomotor level of P5: Complex Overt Response – the ability to skilfully performing complex movements automatically and without hesitation), and demonstrate complex restorative procedures in primary and permanent dentition (Simpson’s psychomotor level of P4: Mechanism - the intermediate stage of skill mastery that entails turning learned responses into habitual reactions) [12, 13]. Consequently, there is a need for Malaysian dental schools to modify their existing paediatric dental curriculum in accomplishing the intended learning outcomes, which would lead to the acquisition of cognitive and psychomotor competencies. This also supports the necessity to determine whether the existing paediatric dental curricula include desired competencies that future dental graduates need to serve the oral health needs of a varied paediatric population in Malaysia.

The first step in advocating a standardised national curriculum for undergraduate paediatric dental education is to identify what topics should be included. The Delphi method may be used to accomplish this since it is a formal consensus approach and a systematic way to gauge and establish expert consensus [14]. The Fuzzy Delphi method is the modified and improved version of the classical Delphi technique [15]. It differs from the traditional approach in that it uses mathematical concepts instead of probability theory [16]. The notion behind combining the conventional Delphi method with fuzzy theories is to improve the ambiguity of qualitative answers among experts [17]. Furthermore, the adoption of the Fuzzy number helps to provide a reliable quantitative approach in eliminating the fuzziness that is often present during the study process and allowing experts to freely share their professional opinions on the topic [18]. Fuzzy Delphi method is also claimed to be superior to traditional Delphi in terms of the number of questions required to achieve expert consensus, the originality of experts’ viewpoints, and the time and cost required to carry out the process due to the need for a small sample size [15]. Hence, the overarching goal of the present study is to identify relevant topics in undergraduate paediatric dental curricula and to determine the level of cognitive and psychomotor necessary for future dental graduates by consensus among experts in the field of paediatric dentistry.

## Methods

The Asian Institute of Medicine, Science and Technology (AIMST) University Human Ethic Committee granted ethical clearance for the current study with the following approval code: AUHEC/FOD/2022/23/11/01.

### Development of the list of relevant topics

Two paediatric dental specialists who are full-time senior faculty members from two different dental schools in Malaysia (Universiti Malaya and Universiti Sains Malaysia) and a private paediatric dental specialist were brought together to form a focus group. They contributed to the development of the first draft of relevant paediatric dentistry topics for the undergraduate dental curriculum by compiling the existing undergraduate dental curricula in each Malaysian dental school through discussion with the heads of department, followed by analysing and comparing them to the revised national competence statement published in 2021. Subsequently, a tentative list of pertinent topics was developed. For the literature review, three databases (Google Scholar, PubMed, and Science Direct) with keywords of “undergraduate paediatric dentistry”, “paediatric dental education”, and “paediatric dental curriculum” were used. The initial list of relevant topics was then modified repeatedly until all three members reached a consensus. The final draft included a list of 65 important topics under 18 specific domains (Restoration in paediatric dentistry; Preventive dentistry in children; Minimal Intervention Dentistry (MID); Dental caries in paediatric patients; Growth and development; Basic behaviour guidance techniques; advanced behaviour guidance technique; Examination and diagnosis in paediatric dentistry; Pulp therapy in primary teeth & immature teeth; First permanent molar; Interceptive orthodontics; Tooth eruption and exfoliation; Trauma in paediatric dentistry; Orofacial soft tissue lesion in paediatric patients; Special care in paediatric patients; Tooth extraction in paediatric patients; Dental materials in paediatric dentistry; and Dental emergencies in paediatric dentistry) and was converted into a survey questionnaire by one investigator, who entered the information into a Google Form survey tool.

### Panel of experts

Content validation was conducted by two paediatric dental specialists (one from the Ministry of Health Malaysia and another from a Malaysian private dental school) to provide a preliminary assessment of the acceptability of the content. No amendment was required. The Fuzzy Delphi method was applied to identify expert opinions in the present study [19]. 25 experts who are currently affiliated to any local dental schools, private sectors or the Malaysian Ministry of Health and fulfilled the inclusion criteria were chosen for the present study, which was performed with the recommended optimum number of experts between 10 and 50 [18]. The inclusion criteria for experts were those with a valid annual practising certificate and fulfilled one of the following: completed a paediatric dental specialist training postgraduate programme or currently enrolled in any postgraduate clinical training programme related to paediatric dentistry science or published in a peer-reviewed paediatric dental journal or actively involved in paediatric dental associations that uphold the best interests of paediatric dental education. Meanwhile, international faculty members were excluded from the current study.

A purposive sampling was used, and the survey items were sent out via an online Google Form to experts from various regions of Malaysia. Each expert is required to score the questionnaire items based on a five-point Likert scale (1 = Not important at all; 2 = Not important; 3 = Neutral; 4 = Important and 5 = Very important) and determine the appropriate cognitive and psychomotor levels using Bloom’s cognitive (1 = remember, 2 = understand, 3 = apply, 4 = analyse, 5 = evaluate, 6 = create) and Simpson’s psychomotor (1 = perception, 2 = set, 3 = guided response, 4 = mechanism, 5 = complex overt response, 6 = adaptation, 7 = origination) taxonomies. The survey was given to the experts with three weeks to complete it, and reminders were sent at the end of the second and third weeks.

### Fuzzy Delphi method

The included experts anonymously assessed the importance of relevant topics that should be covered in the Malaysian undergraduate paediatric dental curriculum. Along with each topic, experts were asked to suggest acceptable cognitive and psychomotor levels. They were also given the freedom to add any new topics that were not on the list. This is followed by the translation of linguistic variables to fuzzy numbers. Three numbers for each recorded answer were used to identify the average lowest value, the fair value, and the highest value represented by *m*1, *m*2, and *m*3, respectively [20]. The *m* values in the present context showed the probability of experts agreeing that the paediatric dentistry topics were important.

Three requirements were used to choose whether to maintain or eliminate topics. First, experts were considered to have reached a consensus on the topics if the difference between the average and expert rating data was ≤ 0.2 [21]. By calculating the difference between each expert’s fuzzy number and the average fuzzy number, the d-construct threshold value for each item was found. To calculate the difference between the averages, the vertex approach was used [22]. Second, a consensus of ˃70% among the experts was acceptable [18], and no further cycle of the survey is needed. Finally, an accepted topic should have an average fuzzy number of ≥ 0.5. A framework of curriculum content in undergraduate paediatric dentistry was then modelled using fuzzy assessments. This stage involved sorting the topics according to the experts’ opinions, with the most important topic in the model receiving the highest value [20].

### Cognitive and psychomotor levels analysis

Bloom’s and Simpson’s levels each received scores ranging from 1 to 6 and 1 to 7, respectively. The average mean scores were calculated using numbers assigned to each topic [23]. The mean ratings were used to establish the appropriate cognitive and psychomotor levels [7], but they did not take into account responses that were deemed as missing.

## Results

20 experts (respondent rate of 80%) who fulfilled the criteria for inclusion agreed to take part in the questionnaire survey. In Table 1, their demographic backgrounds are presented. Most of the experts were affiliated with the Malaysian Ministry of Health and had more than ten years of expertise in the related field. The average fuzzy number for the topics ranged from 0.36 to 0.85, while the average Likert score ranged from 3.05 to 5.00 (Table 2). Since the average fuzzy number for the topic “Dental amalgam” was 0.36 (< 0.5), it was thus rejected based on expert consensus. Meanwhile, the remaining 64 topics were agreed upon by the expert group to be relevant to the undergraduate paediatric dental curriculum. The topic “Pit and fissure sealant” was deemed to be the most important topic, followed by “Preventive advice”, “Early childhood caries”, “Dental caries in children & adolescent”, “Management of dental caries in paediatric patients”, and “Consent” equally ranked as the second most important topics. On the other hand, “Dental amalgam” was ranked as the least important topic, followed by “General anaesthesia”, and “Conscious sedation: Intravenous sedation”. There were no further topics proposed.

**Table 1:** Demographic backgrounds of the fuzzy Delphi experts

Items	respondent (*n*)
**Field of Expertise**	
Paediatric dental specialist	13
Dentist undergoing paediatric dental specialist training programme	7
*Total*	20
**Years of experience**	
Less than 5 years	0
5 to 10 years	11
More than 10 years	9
*Total*	20
**Affiliation**	
Public teaching institution	6
Private teaching institution	3
Malaysian Ministry of Health public hospital / clinic	7
Private hospital / clinic	4
*Total*	20

**Table 2:** Fuzzy Delphi findings on topics and their competency levels for undergraduate paediatric dental curriculum

No	Topic	Average Likert score	Threshold value *d* ≤ 0.2	Expert’s Agreement (%)	Average fuzzy number	Ranking	Verdict	Bloom’s Cognitive Level		Simpson’s Psychomotor Level								
								**Mean score**	**Corresponding level**	**Mean score**	**Corresponding level**							
Restoration in Paediatric Dentistry																		
1	Dental composite resin	4.90	0.055	80	0.78	14	Accept	4.50	Analyse	5.00	Complex overt response							
2	Glass ionomer cement	4.90	0.055	80	0.78	14	Accept	4.50	Analyse	5.00	Complex overt response							
3	Dental amalgam	3.05	0.098	80	0.36	65	Reject	3.60	Apply	3.95	Guided response							
4	Anterior strip crown	4.70	0.137	70	0.74	31	Accept	4.20	Analyse	4.00	Mechanism							
5	Aesthetic crown (zirconia)	3.95	0.137	70	0.62	59	Accept	3.35	Apply	2.85	Set							
6	Stainless steel crown- conventional	4.80	0.104	80	0.76	23	Accept	4.25	Analyse	3.65	Guided response							
7	Tooth isolation technique	4.90	0.061	100	0.78	14	Accept	4.40	Analyse	4.45	Mechanism							
Preventive Dentistry in Children																		
8	Preventive advice	5.00	0.0	100	0.80	2	Accept	4.50	Analyse	4.50	Mechanism							
9	Pit and fissure sealant	4.95	0.029	90	0.85	1	Accept	4.50	Analyse	4.40	Mechanism							
10	Preventive resin restoration	4.95	0.029	90	0.79	7	Accept	4.50	Analyse	4.40	Mechanism							
11	Professionally applied topical fluoride	4.95	0.029	90	0.79	7	Accept	4.60	Analyse	4.45	Mechanism							
Minimal Intervention Dentistry (MID)																		
12	Concept and principle of MID	4.80	0.098	80	0.76	28	Accept	4.45	Analyse	-	-							
13	Resin infiltration technique	4.30	0.150	70	0.66	53	Accept	3.60	Apply	3.30	Guided response							
14	Sodium diamine fluoride (SDF)	4.40	0.165	100	0.68	44	Accept	3.85	Apply	3.70	Guided response							
15	Atraumatic restoration technique	4.80	0.098	70	0.76	23	Accept	4.25	Analyse	4.30	Mechanism							
16	Non-restorative cavity control (NRCC)	4.35	0.159	90	0.67	48	Accept	3.85	Apply	3.80	Guided response							
17	SMART technique	4.30	0.183	90	0.67	48	Accept	3.75	Apply	3.55	Guided response							
18	Stainless steel crown- Hall technique	4.40	0.0	100	0.68	44	Accept	3.75	Apply	3.60	Guided response							
Dental caries in Paediatric Patients																		
19	Early childhood caries	5.00	0.0	100	0.80	2	Accept	4.55	Analyse	4.45	Mechanism							
20	Dental caries in children & adolescent	5.00	0.029	100	0.80	2	Accept	4.60	Analyse	4.05	Mechanism							
21	Caries risk assessment in children & adolescent	4.95	0.0	100	0.79	7	Accept	4.50	Analyse	4.15	Mechanism							
22	Management of dental caries in paediatric patients	5.00	0.151	100	0.80	2	Accept	4.35	Analyse	4.40	Mechanism							
Growth and Development																		
23	General development (birth to adolescent)	4.45	0.115	70	0.69	40	Accept	3.25	Apply	2.95	Set							
24	Developmental child psychology	4.25	0.029	100	0.65	55	Accept	3.30	Apply	2.95	Set							
Basic Behaviour Guidance techniques																		
25	Non-pharmacological behaviour management	4.95	0.126	70	0.79	7	Accept	4.35	Analyse	4.25	Mechanism							
26	Conscious sedation: Inhalation sedation	4.05	0.110	70	0.65	55	Accept	3.45	Apply	2.45	Set							
Advanced behaviour Guidance Technique																		
27	Protective stabilisation	4.25	0.139	100	0.62	59	Accept	2.95	Understand	3.40	Guided response							
28	Conscious sedation: Oral sedation	3.70	0.147	100	0.53	62	Accept	2.85	Understand	2.50	Set							
29	Conscious sedation: Intravenous sedation	3.60	0.151	100	0.52	63	Accept	2.75	Understand	1.95	Perception							
30	General anaesthesia	3.55	0.0	100	0.51	64	Accept	2.60	Understand	1.95	Perception							
Examination and Diagnosis in Paediatric Dentistry																		
31	Consent	5.00	0.055	80	0.80	2	Accept	4.50	Analyse	5.00	Complex overt response							
32	History, Examination, Diagnosis, Treatment Planning and Recalls	4.90	0.029	90	0.78	14	Accept	4.65	Analyse	5.00	Complex overt response							
33	Radiographs for Paediatric Dental Patients	4.95	0.078	80	0.79	7	Accept	4.70	Analyse	4.60	Mechanism							
Pulp Therapy in Primary Teeth and Immature Teeth																		
34	Pulp pathology & deep caries management	4.85	0.165	100	0.77	19	Accept	4.35	Analyse	4.20	Mechanism							
35	Apexogenesis and apexification	4.40	0.128	80	0.68	42	Accept	3.35	Apply	2.85	Set							
36	Vital pulp therapy	4.70	0.115	70	0.74	31	Accept	4.10	Analyse	3.95	Guided response							
37	Lesion sterilisation and Tissue repair (LSTR) in primary teeth	4.10	0.139	100	0.65	55	Accept	3.25	Apply	3.15	Guided response							
38	Non-vital pulp therapy in primary teeth	4.65	0.165	90	0.73	35	Accept	4.10	Analyse	3.90	Guided response							
39	Non-vital pulp therapy in immature permanent teeth	4.40	0.147	100	0.68	44	Accept	3.40	Apply	3.15	Guided response							
40	Non-vital pulp therapy in mature permanent teeth	4.60	0.078	90	0.72	37	Accept	3.65	Apply	3.40	Guided response							
First Permanent Molar																		
41	Importance of first permanent molars	4.85	0.137	80	0.77	19	Accept	4.35	Analyse	-	-							
42	Management of compromised first permanent molars	4.70	0.153	100	0.74	33	Accept	3.95	Apply	3.30	Guided response							
43	Molar Incisor Hypomineralisation	4.70	0.122	70	0.74	33	Accept	3.90	Apply	3.45	Guided response							
Interceptive Orthodontics																		
44	Interceptive orthodontics concepts	4.50	0.168	90	0.70	39	Accept	3.40	Apply	-	-							
45	Space maintenance	4.20	0.159	90	0.64	58	Accept	3.40	Apply	2.90	Set							
Tooth Eruption and Exfoliation																		
46	Developmental and acquired disturbances in teeth	4.45	0.165	90	0.69	40	Accept	3.50	Apply	2.80	Set							
47	Inherited disorders and syndromes associated with tooth anomalies	4.35	0.179	90	0.67	47	Accept	3.50	Apply	2.70	Set							
48	Problem associated with eruption and exfoliation of teeth	4.40	0.098	70	0.68	42	Accept	3.55	Apply	2.80	Set							
49	Management of dental defects	4.35	0.098	70	0.67	52	Accept	3.30	Apply	3.40	Guided response							
Trauma in Paediatric Dentistry																		
50	Trauma to permanent teeth and its management	4.80	0.098	70	0.76	23	Accept	4.05	Analyse	3.75	Guided response							
51	Trauma to primary teeth and its management	4.80	0.115	100	0.76	23	Accept	4.05	Analyse	3.70	Guided response							
52	Avulsed permanent teeth and its management	4.80	0.139	100	0.76	23	Accept	4.05	Analyse	4.05	Mechanism							
53	Orofacial soft tissue injuries	4.75	0.128	80	0.75	29	Accept	3.95	Apply	3.70	Guided response							
54	Child abuse and neglect / non-accidental injury / safeguarding in children	4.55	0.087	80	0.71	38	Accept	3.25	Apply	2.10	Set							
Orofacial Soft Tissue Lesions in Paediatric Children																		
55	Types of soft tissue lesion	4.35	0.115	70	0.67	48	Accept	3.45	Apply	-	-							
56	Oral manifestation in systemic diseases	4.30	0.139	100	0.66	54	Accept	3.30	Apply	2.70	Set							
57	Management of soft tissue lesion	4.05	0.029	90	0.61	61	Accept	3.05	Apply	3.05	Guided response							
Special Care in Paediatric Patients																		
58	Management of medically compromised children	4.75	0.029	90	0.75	29	Accept	3.35	Apply	3.10	Guided response							
59	Management of special needs children	4.65	0.055	90	0.73	35	Accept	3.35	Apply	3.05	Guided response							
Tooth Extraction in Paediatric Patients																		
60	Local anaesthesia in children	4.95	0.179	90	0.79	7	Accept	4.20	Analyse	4.70	Mechanism							
61	Exodontia in children	4.95	0.078	80	0.79	7	Accept	4.20	Analyse	4.75	Mechanism							
62	Post-operative management and complications	4.90	0.078	80	0.78	14	Accept	4.20	Analyse	4.60	Mechanism							
Dental Materials in Paediatric Dentistry																		
63	Current and advanced materials	4.35	0.179	90	0.67	48	Accept	3.05	Apply	2.60	Set							
Dental Emergencies in Paediatric Dentistry																		Accept
64	Acute odontogenic infections	4.85	0.078	80	0.77	19	Accept	4.15	Analyse	3.25	Guided response							
65	Acute pain management	4.85	0.078	80	0.77	19	Accept	3.90	Apply	3.20	Guided response							

-: Not Applicable

The mean scores for the cognitive and psychomotor levels according to Bloom’s and Simpson’s taxonomies, are shown in Table 2 accordingly. 31 paediatric dentistry topics were rated adequate for undergraduate students at the cognitive level of “Apply” (C3), followed by 30 topics at the cognitive level of “Analyse” (C4), and the remaining four topics at the cognitive level of “Understand” (C2). The lowest cognitive level of “Remember” (C1), as well as the higher cognitive levels of “Evaluate” (C5) and “Create” (C6), were generally not taken into consideration by experts. On the contrary, 24 topics were found suitable for dental students to achieve the psychomotor level of “Guided response” (P3), followed by 17 topics with a psychomotor level of “Mechanism” (P4). 13 topics were considered appropriate at the psychomotor level of “Set” (P2), four topics at a higher psychomotor level of “Complex overt response” (P5), whereas two topics were only deemed appropriate to attain the lowest psychomotor level of “Perception” (P1). Four topics were not relevant to identify the psychomotor level which were “Concept and principle of MID”, “Importance of first permanent molars”, “Interceptive orthodontics concepts”, and “Types of soft tissue lesions”. The highest levels of “Adaptation” (P6) and “Origination” (P7) are not necessary for undergraduate students according to experts’ consensus based on mean scores. Nonetheless, the current findings met the desired cognitive and psychomotor levels listed in the revised national competency statement relevant to paediatric dentistry.

## Discussion

The present study focused on the usage of fuzzy Delphi method to gather expert opinion in identifying relevant paediatric dentistry topics for undergraduate dental curriculum and determining the suitable cognitive and psychomotor levels for each topic. Moreover, it is the first of its kind to model a framework for undergraduate paediatric dental curricula in Malaysia. Since the quantification of expert opinion about a particular subject of discussion cannot accurately reflect human thought processes when applying the traditional Delphi approach, fuzzy Delphi was employed in the present study as the decision-making based on the fuzzy set is claimed to be more reliable [24].

Throughout the consensus-building among 20 experts in the present study, the topic of “Pit and fissure sealant” was ranked as the most relevant topic for the Malaysian undergraduate paediatric dental curriculum. Such a finding is consistent with previous studies [8, 25], highlighting that the most essential clinical training programme for dental undergraduate students is preventive dentistry as caries prevention is the foundation of the specialisation. Similar findings were also reported in previously published works highlighting that most universities in the United Kingdom and Brazil emphasise the knowledge and application of fissure sealants in undergraduate dental education [9, 26]. Undeniably, pit and fissure sealants are the most effective way to reduce occlusal caries [27], and have been used for over five decades to prevent and treat carious lesions on both primary and permanent teeth [28]. Furthermore, sealant material micromechanically bonds to the tooth and prevents cariogenic bacteria from accessing their source of nutrients. Nevertheless, recent research on Malaysian dental practitioners revealed that less than 20% of them used pit and fissure sealants in their routine clinical practises, and only 57.5% of the practitioners were aware of the guidelines for fissure sealant application [29]. It is thus not astonishing that “pit and fissure sealant” was regarded as the most significant topic in the current study given the importance of maintaining the appropriate practice of such a preventative strategy in halting caries development among children.

Five topics were equally ranked as the second most significant topic for undergraduate paediatric dentistry with the majority falling under the domain of “Dental caries in paediatric patients”. One of the most prevailing oral diseases that could be prevented among children is dental caries [30], and future dental graduates should be well-trained in minimising cavities among paediatric patients cost-effectively. It is worth noting that the progression of dental caries and its management in children require a deeper knowledge and dental graduates are required to detect and treat paediatric dental caries with proper preventive advice that fall within their scope of expertise [31]. The importance of dental caries among children can also be linked to the high prevalence of dental caries among preschool children in Malaysia. “Consent” is also ranked as the second most relevant topic and this is critically important as informed consent must be sought and recorded properly in patient’s treatment folder before starting any dental procedures since it is the foundation for developing trust and respect between the paediatric patient and the dental practitioners [32, 33]. Apart from that, dental students should be trained on how to obtain valid consent and who can give consent for a paediatric patient.

Based on the present findings, “Dental amalgam” was the only topic removed from the list. The International Association for Dental Research has stated that apart from situations when no other dental restorative materials are available, dental amalgams should be phased out by 2024 despite being used as restorative materials for many years [34]. However, a study conducted in the Arabian Peninsula found that most dental programmes still include theoretical and practical guidance on amalgam restorations [8]. In contrast, several countries such as Sweden, Norway, Denmark and Germany have taken initiatives to gradually reduce the use of dental amalgam or even banned it [35]. Besides, a statement on the phase-down of dental amalgam was released by the Minamata Convention on Mercury, a worldwide accord aimed at reducing anthropogenic emissions and releases of mercury [36]. These could be the factors “dental amalgam” did not reach a consensus in the current study. In addition, the Minamata Convention on Mercury and the FDI-World Dental Federation’s attempts to gradually phase out the use of dental amalgam were supported by the Malaysian Dental Council, which explained its stance in a position statement [37].

“General anaesthesia” and “Conscious sedation: Intravenous sedation” were ranked as the second and third least important topics for undergraduate paediatric dental curricula. Similarly, these two topics received the lowest psychomotor level (P1) according to expert opinions. Whilst conscious sedation and general anaesthesia were commonly taught in the undergraduate paediatric dental programmes in the United Kingdom, United States and Canada [38, 39], teaching of these techniques appeared to be less favourable in Malaysia. Due to the intricacy of these behavioural management techniques and the requirement for specialised training, only fundamental knowledge is being taught in most Malaysian undergraduate dental curricula via lectures with no hands-on experience. This could plausibly be the reason “Advanced Behaviour Guidance techniques” domain received a lower cognitive level (C2) based on expert consensus. Moreover, there are presently no mandatory training requirements in Malaysia for dental practitioners to safely administer conscious sedation, nor are there any comprehensive national recommendations on how to manage children under conscious sedation. It is therefore difficult to foresee that these advanced behaviour guidance techniques would be covered in Malaysian undergraduate paediatric dental curricula. Nonetheless, dental graduates commonly find themselves in a position to refer patients to specialised paediatric care and knowing these advanced behaviour guidance techniques will help ensure that the referral is made appropriately [9]. Thus, dental educators may consider including more training, or at least providing opportunities for students to observe or assist in conscious sedation and general anaesthesia procedures.

Given that Malaysia is transitioning its undergraduate dental curricula into competency-based dental programmes that are in line with the national competency statement, it is essential that dental students should have adequate cognitive and psychomotor levels on the fundamental principles of paediatric dentistry. There is a discernible trend where topics under the domains “Preventive Dentistry in Children”, “Dental Caries in Paediatric Patients”, “Examination and Diagnosis in Paediatric Dentistry” and “Tooth Extraction in Paediatric Patients” were ranked at a higher cognitive level. Meanwhile, four topics, “Dental composite resin”, “Glass ionomer cement”, “Consent” and “History, Examination, Diagnosis, Treatment Planning and Recalls” were concurred to reach a higher psychomotor level by experts. It is reasonable to argue that dental students should be competent in obtaining valid consent, performing history taking and physical examination with accurate diagnosis and treatment planning, mixing and placing glass ionomer and dental composite resin in paediatric patients which are in accordance with the findings from undergraduate dental training in the United Kingdom [9]. Moreover, glass ionomer cement and dental composite resin are regarded as the most commonly used restorative materials in restoring primary and permanent dentitions among paediatric patients in Malaysia [40]. Interestingly, “Aesthetic crown (zirconia)” ranked at a lower psychomotor level (P2: set) as compared to other restorations in paediatric dentistry which might be due to the relative complexity of the technique, the amount of dental preparation needed, and the potential financial consequences [9].

One strength of the present study is that it can serve as a standard for all Malaysian public and private dental schools as they tailor their current undergraduate dental programmes per the revised national competency statement. The use of fuzzy Delphi mathematical analysis can also help to avoid the intervals and ambiguous meaning limits of the Likert scale [41]. Most importantly, the present method offers a suitable quantitative approach to typical qualitative group discussions or gatherings [20]. Nevertheless, one of the study’s drawbacks is that experts need to be reminded frequently to give their responses, which might result in emotional bias [20]. It is also crucial to note that dental schools from other countries might not agree with the present undergraduate paediatric dentistry topics. It is conceivable that international dental schools have their own programme learning outcomes and educational standards that are more adapted to the requirements of their unique populations. The recommended relevant paediatric dentistry topics for undergraduate dental curricula, however, may only be considered a prototype as they have not been implemented among Malaysian dental students. Thus, more research is necessary to determine its relevance and effectiveness in undergraduate dental programmes.

## Conclusion

The relevance of paediatric dentistry topics has been successfully identified in the present study using the fuzzy Delphi method which can aid future educators in improving existing undergraduate paediatric dental curricula. Expert opinions indicated that “Dental amalgam” is to be removed, while the remaining 64 topics were accepted. Moreover, “Pit and fissure sealant” was ranked as the most important topic. Most of the topics were considered appropriate at the psychomotor level of “Guided response” and cognitive level of “Apply”. To guarantee their applicability and efficacy in undergraduate dental curricula across the country, the identified relevant paediatric dentistry topics still need additional reliability and validity evaluation and pilot testing. Before implementation across all dental schools in Malaysia, specific learning objectives, pedagogical, and assessment strategies are required, given the development of the current pertinent undergraduate paediatric dentistry topics.

## Data Availability

All data generated or analysed during this study are included in this published article.

## Abbreviations

AIMST: Asian Institute of Medicine, Science and Technology University
MDC: Malaysian Dental Council
MID: Minimal Intervention Dentistry
MQA: Malaysian Qualifications Agency
